# Diaphragm Training in Patients With Left Ventricular Assist Devices: A Narrative Review of Rehabilitation and Clinical Implications

**DOI:** 10.7759/cureus.100171

**Published:** 2025-12-27

**Authors:** Bruno Bordoni, Bruno Morabito, Allan R Escher

**Affiliations:** 1 Physical Medicine and Rehabilitation, Foundation Don Carlo Gnocchi, Milan, ITA; 2 Physical Medicine and Rehabilitation, School of Osteopathic Centre for Research and Studies, Milan, ITA; 3 Oncologic Sciences, University of South Florida Morsani College of Medicine, Tampa, USA; 4 Anesthesiology/Pain Medicine, H. Lee Moffitt Cancer Center and Research Institute, Tampa, USA

**Keywords:** american college of cardiology, american heart association, cardiac rehabilitation, diaphragm, heart failure society of america, inspiratory muscle training, interagency registry for mechanically assisted circulatory support (intermacs), left ventricular assist devices, lvad, physiotherapy

## Abstract

Chronic heart failure (HF) is a syndrome that affects millions of people globally each year. If a patient with HF does not respond to standard therapy (pharmacological and/or instrumental), the gold standard treatment for end-stage HF is heart transplantation (HTx). The increasing number of end-stage HF patients does not correspond to the number of available HTx procedures. To address this discrepancy and attempt to prolong life, another therapeutic option is the implantation of durable left ventricular assist devices (LVADs), with the next-generation HeartMate 3 device (HM3; Chicago, IL: Abbott). As with HF, LVAD patients can safely access a cardiac rehabilitation (CR) program, with various clinical benefits and improved quality of life. What emerges from the literature is the near-absence of guidance on diaphragm rehabilitation, despite the pathophysiological adaptations this muscle undergoes. This review aimed to emphasize the importance of routinely incorporating inspiratory muscle training (IMT) into the CR process. The article will also briefly review the history of LVADs, with a focus on newer generations of devices, including how the device works, the adverse events that occur, and the positive clinical adaptations observed with CR.

## Introduction and background

Chronic heart failure (HF) is a syndrome that affects millions of people globally every year. In China, there were approximately 4.5 million patients with heart failure (HF) in 2020, while the total number of HF patients across Asian countries was estimated at 31.89 million in 2019. In Canada, HF affects approximately 3.5% of the population. In the United States, projections estimate that more than eight million individuals will have HF by 2030, representing approximately 3.9% of the population [1-4]. In Europe, there is considerable heterogeneity in the data, with prevalence estimates ranging from a minimum of 1.4% of the population in Italy to a maximum of 2.2% in Sweden [5]. One to three percent of the world's population is diagnosed with HF, with the death rate within one year of diagnosis being approximately 15-30% [6-8].

If the patient with HF does not respond to pharmacological therapy, instrumental therapy (pacemaker, implantable cardioverter defibrillator), and physical activity (cardiac rehabilitation), the gold standard treatment for end-stage HF (10% of the HF population) is heart transplantation (HTx) [7,9]. The increase in the elderly population clashes with the reduced availability of HTx. To address the discrepancy between the demand for and shortage of heart transplantation (HTx), and to extend survival in extremely clinically fragile patients, durable mechanical circulatory support (DMCS) device technology has advanced significantly, providing clinicians with expanded therapeutic options. The most commonly implanted devices are durable left ventricular assist devices (LVADs) [10,11].

The latest guidelines from the International Society for Heart and Lung Transplantation (ISHLT) define the patient populations eligible for LVAD implantation. Patients with HF and New York Heart Association (NYHA) functional classification IIIB-IV (level of evidence A), with a left ventricular ejection fraction (LVEF) ≤30%, are indicated for LVAD implantation; consideration is also given to patients with newly diagnosed, dilated cardiomyopathy of non-ischemic etiology, refractory to pharmacological therapies and maximal medical therapy (level of evidence B) [12,13].

Based on risk stratification and clinical history, the Interagency Registry for Mechanically Assisted Circulatory Support (INTERMACS), and the European Registry for Patients with Mechanical Circulatory Support (EUROMACS), patients with LVADs are categorized according to similar characteristics as follows: bridge to transplant (BTT), referring to patients with a high percentage of death before transplantation; bridge to candidacy (BTC), referring to patients implanted to improve the systemic picture before transplantation; and destination therapy (DT), referring to patients who do not meet the clinical requirements to undergo transplantation [14-16]. Other patient categories include bridge to decision (BTD) or bridge to bridge (BTB), referring to patients implanted because they are at immediate risk of death, and bridge to recovery (BTR), which includes patients with a high likelihood of device explantation following effective cardiac recovery [15,16].

Currently, the most implanted LVAD model is the HeartMate 3 (HM3; Chicago, IL: Abbott), which demonstrated a 38% five-year post-operative mortality rate in 485 patients (data from the Multicenter Study of MagLev Technology in Patients Undergoing Mechanical Circulatory Support Therapy with HeartMate 3 {MOMENTUM 3} trial) [17]. Compared with previous LVAD implants in the years 2018-2022, HM3 (for a total of 10,920 patients) demonstrated a higher one-year survival rate of 86% and a 64% higher five-year survival rate, according to data from INTERMACS [18]. In these data, the percentage of female patients with implants was 22%, whereas data from the MOMENTUM 3 trial (in the European area) indicated that male patients had an implantation rate of 83.6% [17,18].

Cardiac rehabilitation (CR) is an effective and proven tool to improve several clinical and functional aspects and the quality of life of patients with HF, following physical activity (aerobic activity/endurance training, resistance training), and inspiratory muscle training (IMT) [19,20]. CR for patients with LVAD is safe and effective, resulting in a decrease in mortality and rehospitalization. In a single-center cohort (138 patients participated in CR), patients participating in CR gained a 96% survival rate at one year after implantation, compared with patients who did not follow rehabilitation [21,22].

What emerges from the literature is the limited importance given to IMT in a CR program for patients with LVADs. Furthermore, the diaphragm is an underappreciated muscle in clinical practice in this patient population. This narrative review aimed to emphasize the importance of routinely adding IMT to rehabilitation programs, and to give a more "heavy" clinical look at the diaphragm. This article will also briefly review the history of LVADs, with a focus on newer generations of implants, including their functioning, adverse events, and the positive clinical adaptations observed with CR. Articles were selected for inclusion based on their relevance and publication date.

## Review

Historical Path of LVADs

Knowing the history of the LVAD, how the machine works, and adverse events (as discussed further in this article) enables clinicians and physiotherapists to understand how to manage patients in a rehabilitation setting. The first implantation of a primitive LVAD (pneumatically operated pump) was performed for acute post-cardiac surgery (post-cardiotomy shock) in 1963, and as a bridge to recovery (BTR); the patient died after four days due to systemic complications [23]. Another successful implantation was performed in 1966, again for post-cardiotomy shock and for BTR; the patient managed to survive for 10 days post-implantation (pneumatically operated pump), before the pump was removed [24].

In 1969, a mechanical device with two reciprocating pumps, pneumatically operated for 64 h, and for bridge to transplant (BTT) was implanted; the patient died before heart transplantation [25]. In 1975, an abdominal LVAD (ALVAD) was successfully implanted for BTT [26]. In 1981, a total artificial heart was implanted for BTT, using two pneumatic pumps, still of cumbersome dimensions [27]. The following year saw the first DT implant, with the patient surviving for approximately four months [28]. In 1985, LVADs began to be implanted more safely for BTT, although the machines remained cumbersome [29].

**Figure 1:**
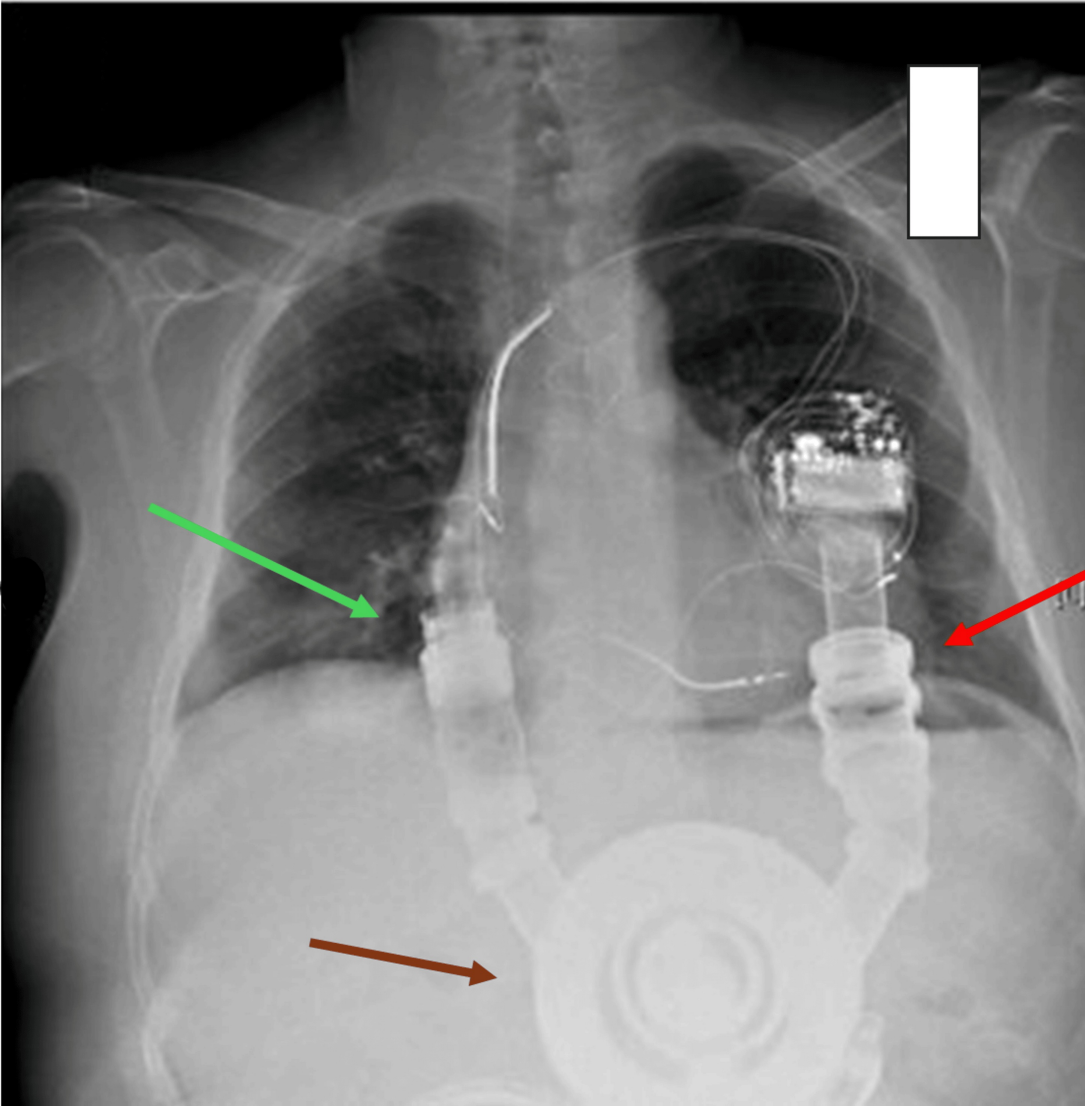
The X-ray shows a patient with a first-generation HeartMate 1 LVAD implant; it is larger than the third-generation model. The drive line is not visible on the X-ray, which was connected to an external controller; this controller connected the external batteries. Red arrow: inflow-valve housing; green arrow: outflow-valve housing; brown arrow: prosthetic left ventricle. This image was provided by the author (Bordoni Bruno) of this article. LVAD: left ventricular assist device HeartMate 1 (HM1; Chicago, IL: Abbott)

The 1990s-2000s saw the birth of the first pulsatile LVADs, such as the Thoratec implantable/paracorporeal VAD (IVAD/PVAD), including the HeartMate, for the treatment of intractable HF patients [15]. This first generation attempted to mimic cardiac pulsation with pulsatile flow but had multiple shortcomings (short-lasting batteries, size, non-resilient materials), resulting in hemorrhages, high rates of thrombosis, and infections, with functional limitations for the patient (Figure 1) [15,30].

LVAD: Second Generation

**Figure 2:**
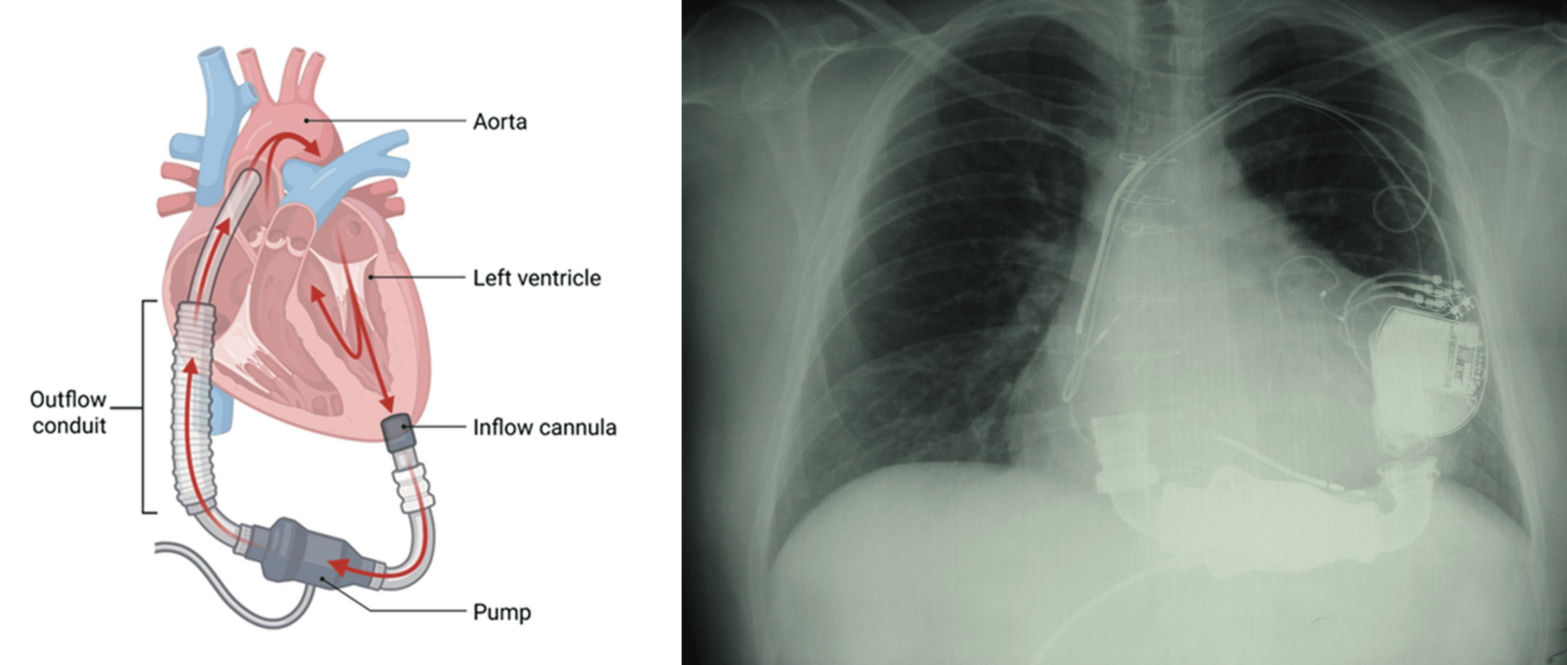
The image shows the second generation of HeartMate (HM). On the left, a drawing representing HM2, while on the right, the X-ray shows a patient with an HM2 implant, which is smaller than the HM1. This illustration was created by the author (Bordoni Bruno) of this article using BioRender.com. HeartMate 2 (HM2; Chicago, IL: Abbott)

The second generation (HeartMate 2 and Jarvik 2000 {New York City, NY: Jarvik Heart, Inc.}) was designed for continuous-flow operation, a smaller size, and more durable materials. This technological adaptation enabled patients to have a greater survival and independent mobility; the device consisted of a single axial internal rotor [15,30]. A 2007 study of second-generation LVADs used for BTT, including 133 patients (20% women), reported survival rates of 71% at six months and 68% at 12 months post-surgery [31]. In 2009, another study compared the first two generations with DT, of which 134 patients had continuous flow and 66 patients had pulsatile flow. The survival rate of patients with continuous flow was 46% at two years after implantation, whereas only 11% for the second group [32]. A major limitation of the second-generation LVADs was the need for continuous anticoagulant therapy, required because of direct contact between the rotor and blood to prevent thrombus formation. Furthermore, the survival rate of these patients did not exceed that of patients with HTx (Figure 2) [15,32].

LVAD: Third Generation

**Figure 3:**
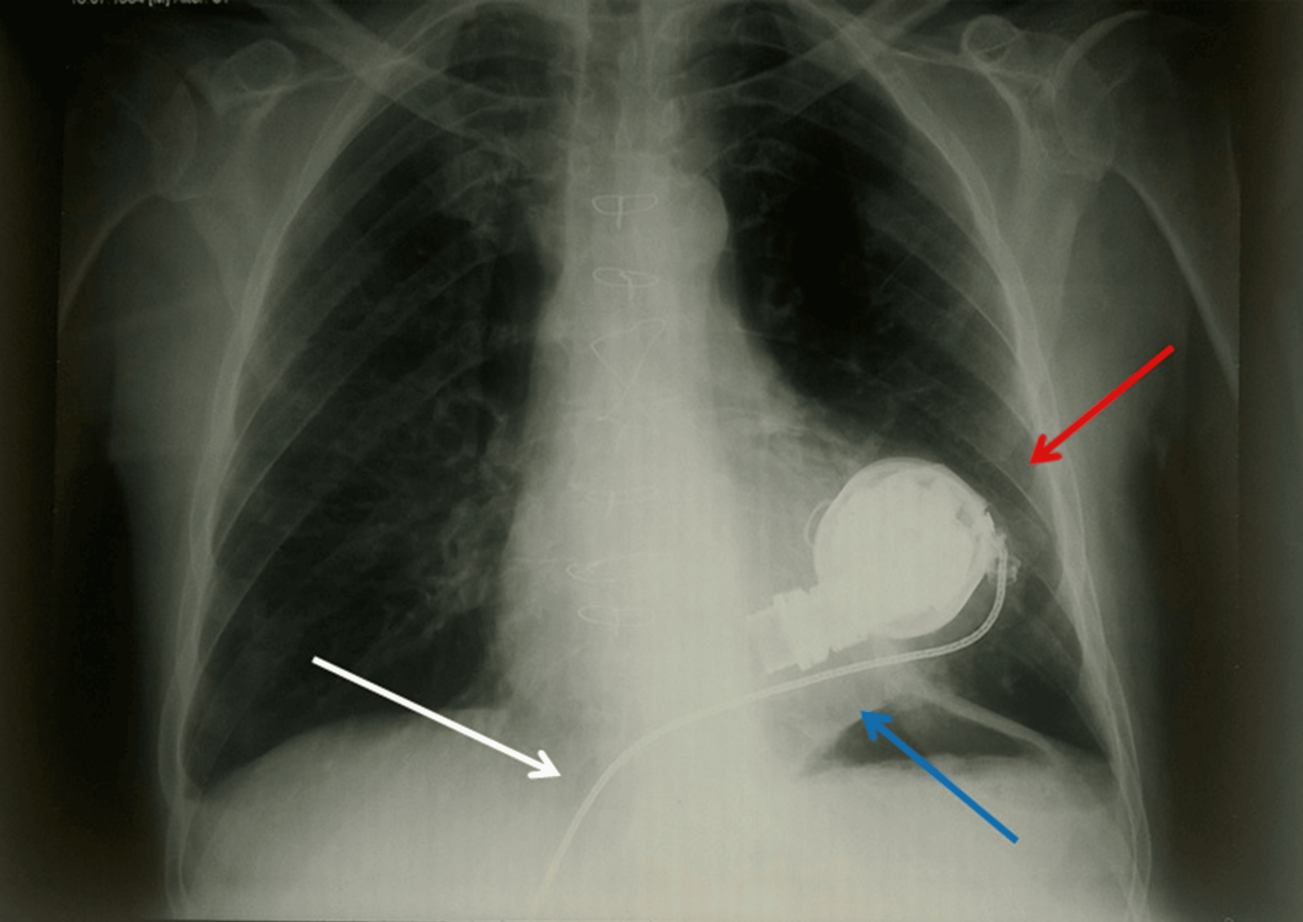
The X-ray shows the HM3 implantation at the apex of the left ventricle. The size is significantly smaller than previous generations, with less invasive implantation. Red arrow: magnetic levitation pump; blue arrow: left ventricle; and white arrow: drive line. This image was provided by the author (Bordoni Bruno) of this article. HeartMate 3 (HM3; Chicago, IL: Abbott)

The third generation of continuous-flow devices (HeartMate 3 and HeartWare Ventricular Assist Device or HVAD {Framingham, MA: HeartWare, Inc.}) allowed for a smaller, more hemocompatible device with a reduced rate of thrombosis. These LVADs use a magnetic levitation system (HM3) or a hybrid magnetic/hydrodynamic system to minimize friction between the blood and the pump [15,32]. HM3 and HVAD weigh 220 g and 160 g, respectively, and operate at a centrifugal speed of 10 L/min; only HM3 can generate an artificial pulsation by varying the rotor speed (Figure 3) [30,33]. HVAD was withdrawn from the market in 2021.

HM3 has demonstrated a six-month survival rate of 92%, 82% at one year, and 76% at two years in 50 patients on DT and BTT, with no thrombosis or device failure events [17,34-36]. Patients with HM3 (according to 2023 INTERMACS data) demonstrate a five-year survival rate of 59.7%, compared to 43.7% for HM2 [11]. Data from MOMENTUM 3 (ELEVATE registry) from 2023 reported a five-year survival rate of 63.3% [15]. The highest mortality rate is observed in patients over 75 years of age (66%), while it decreases in younger age groups, 54% for those aged 65-75 years and 34% for patients under 65 years [15].

Current data indicates that patients undergoing LVAD implantation for BTT are in a lower percentage (18.9% in 2018 compared to under 4% in 2023), and with a decrease in implants for BTC (24.5% in 2018 compared to 14.9% in 2023), where the highest percentages are recorded for patients with DT (81.4% in 2023) [11].

How HM3 works

**Figure 4:**
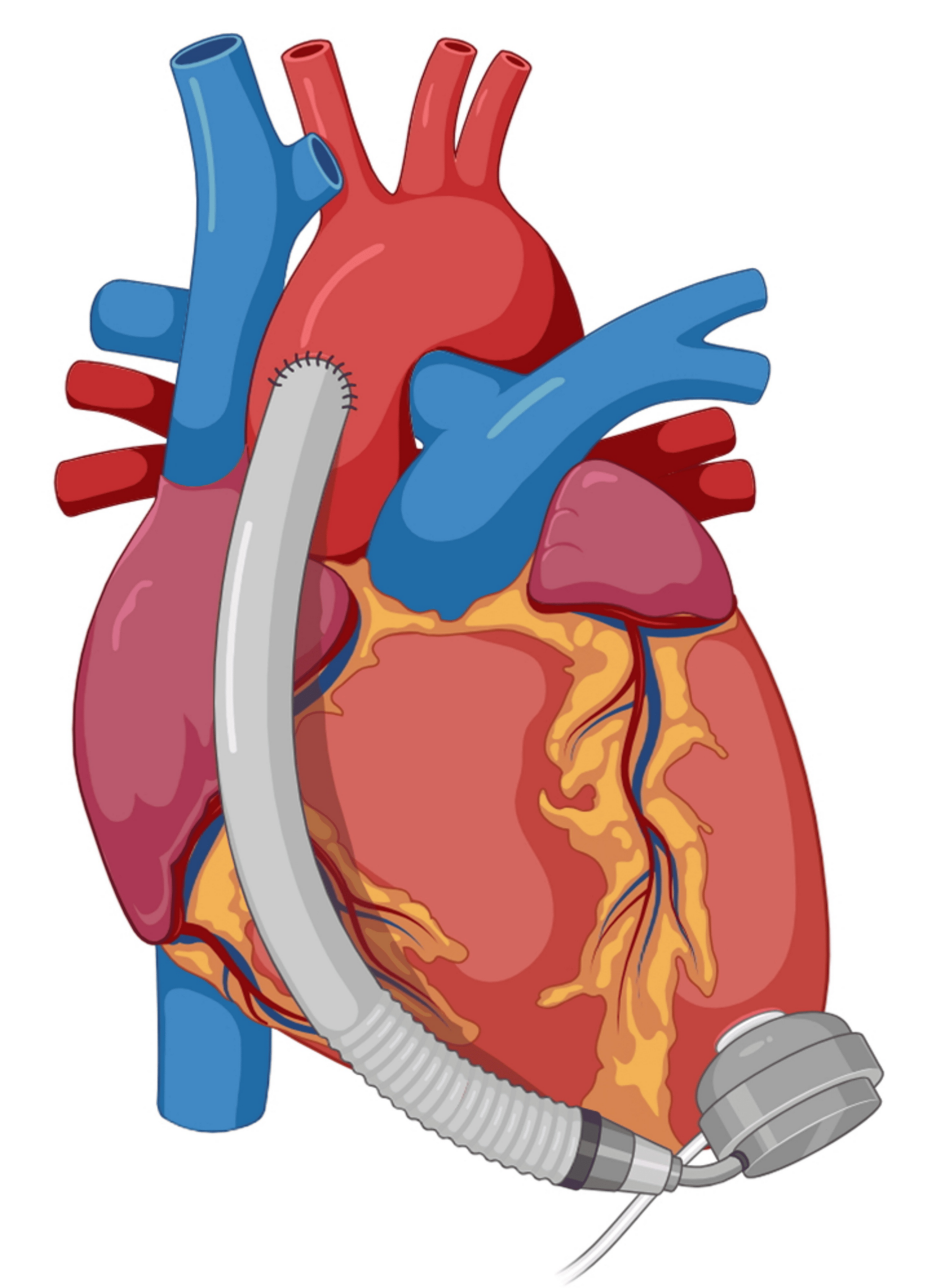
The drawing shows the apex of the left ventricle with the positioning of the inflow cannula and the pump; the driveline and the outflow cannula for the ascending aorta exit from the pump. The model used is an HM3. This image was created by the author (Bordoni Bruno) of this article using BioRender.com. HeartMate 3 (HM3; Chicago, IL: Abbott)

The first HM3 was used on a patient in 2014, and since 2021, it has been the only device implanted [37,38]. Generally, patients undergo cardiac surgery via median sternotomy, or, to a lesser extent, with a left and anterolateral approach (6%), or with a double mini-thoracotomy (10%) [8,39]. The apex of the left ventricle and the pericardium are opened (apical ventriculotomy) The most frequent infectionsto position the inflow sewing ring, and then the inflow cannula is introduced, which is connected to the pump (monitored by transesophageal ultrasound); the pump's outflow cannula is connected to the descending aorta [12]. The pump is connected to the controller via the driveline. The driveline is tunneled from under the diaphragm muscle and through the fascia and rectus abdominis muscle tissue, between the costal margin (right or left) and the umbilicus [12]. The silicone of the driveline is in contact with the skin. Rechargeable batteries are in communication with the controller. The driveline consists of two sets of three wires each: one wire transmits information about the pump’s operation, which can be read by the clinician on a display, and two wires provide power to the pump. One set of wires serves as a spare (Figure 4) [40].

The Magnetic Levitation Pump

The magnetic levitation pump with programmable speed allows for avoiding excessive shear stress on the blood flow, while the titanium microspheres present in the pump interface with the endothelial tissue, with the aim of avoiding the formation of clots [38,41]. Furthermore, approximately every two seconds there will be a sudden decline in the rotor revolutions (for example, two thousand revolutions per minute), with a decrease in speed of approximately 0.15 s and a recovery of speed for approximately 0.25 s (for example, four thousand revolutions per minute), to then return to the speed set by the clinician (for example, six thousand revolutions per minute). This allows for automatic "washing" of the pump and a further reduction in the possibility of thrombus formation.

The Controller Display

From the controller display, the clinician can control and interact with the pump parameters. The HM3 pump speed must be between 5,000 and 6,000 revolutions per minute (RPM). The speed variation for this device and to remain within clinical safety parameters should be within one hundred RPM [40]. The wattage, which reflects the pump power, can be seen and must be between 4.5 and 6.5 watts. The pump flow, measured in liters of blood per minute, can be observed and is determined by the pump speed and power, reaching a maximum of 10 L/min under stress.

The Pump Flow

**Figure 5:**
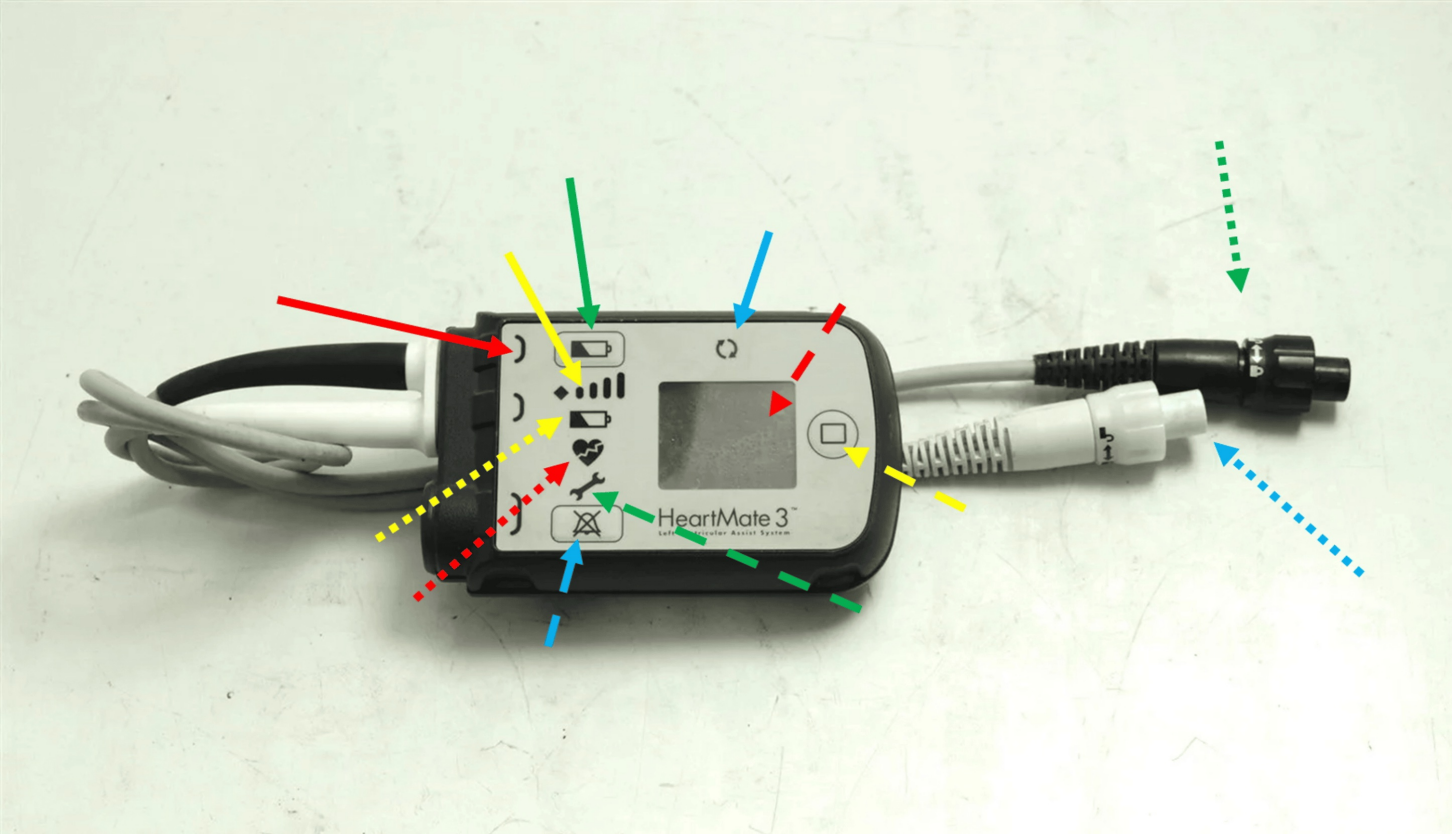
The photo shows a controller model for HeartMate3. The interface displays the following various parameters: the battery button, to control the machine's power; the silence alarm button, to silence alarms and to control previously reported alarms; the pump running light, to assess whether the pump is functioning properly; the display button, for general assessment of the pump; the battery capacity light, to indicate how much power the pump is delivering, useful for managing the batteries; the low battery alert, which warns when the pump is running out of power in a few minutes; the cable disconnect symbols, when the first symbol lights up yellow, indicates a problem with the connection cables, while if the second symbol lights up red, there is no connection to the controller; the yellow wrench, which lights up when the controller is experiencing some kind of malfunction; the hazard alarm, which lights up when there is a serious problem, requiring medical attention; the power cables, for the batteries; and the driveline connector, which comes from the pump. Red arrow: cable disconnect symbols; yellow arrow: battery capacity light; green arrow: battery button; blue arrow: pump running light; dotted red arrow: interface; dotted yellow arrow: display button; dotted green arrow: yellow wrench; dotted blue arrow: silence alarm button; dotted red arrow: hazard alarm; dotted yellow arrow: low battery alert; dotted green arrow: power cables; dotted blue arrow: driveline connector. HeartMate 3 (Chicago, IL: Abbott)

Pump flow is indirectly related to the pressure difference between the inflow and outflow cannulas, and directly to the rotor speed [40]. The flow pulse is determined by the pulsatility index (PI). PI is influenced by pump speed and left ventricular residual contractility. PI is indicated by a number and a time-averaged waveform; PI is an important value to understand the effectiveness of the pump. High numbers are interpreted as greater filling of the left ventricle (preload) and higher pulsatility of the same ventricle, while low numerical values ​​indicate the exact opposite [40]. Low PI may indicate pump obstruction, which is why PI is a value that should not fluctuate excessively during the day and should be monitored on a regular basis (Figure 5) [40]. A correct PI value for HM3 should be between 3.5 and 5.5 [40]. The LVAD is a complex device that interfaces with both the clinician and the patient, allowing rapid intervention in case of an emergency.

Major adverse events

Despite improvements in technology with HM3, patients are not exempt from adverse events, which increase hospital readmissions; 70% of patients require medical care within one year of implantation [10]. Following LVAD implantation, patients may experience right ventricular dysfunction (RHF) or "late right heart failure" (after one month), with rates ranging from 8.3% (in an evaluation of 48 patients) to 45% (in an evaluation of 183 patients), with one-sixth of these patients presenting with concomitant RHF and aortic insufficiency (AI) at six months post-implantation [10,12,42,43]. The finding of RHF is associated with a high mortality prognosis [43]. The origin of this dysfunction may derive from pre-operative conditions, intra-operative complications, and/or the action of the LVAD itself. The latter increases the preload of the right ventricle, which may not be able to adapt to the new condition of increased flow [43]. The MOMENTUM 3 trial highlights that five years after implantation, 29.9% of patients report RHF (29.8% at six months, 27.9% at two years) [43].

The National Inpatient Sample and the National Readmission Database in the United States are the largest and most up-to-date databases on issues that bring patients back to the hospital. From these data, in the period 2016-2022, it emerges that patients with LVAD (92.7% receiving HM3) after at least 30 days return to the hospital more frequently for HF [44]. The percentage of rehospitalization for HF can reach 24% [45]. HF is the major cause of death, with a percentage of 14.2% in a population of 10,920, and LVAD with a new generation pump [18,43].

Another adverse event is bleeding. One study found that patients undergoing HM3 implantation had rehospitalization for bleeding within approximately two years of surgery, with a rate of 13.2% out of 182 patients [46]. Patients older than 75 years have a higher risk of gastrointestinal bleeding [15]. INTERMACS reported a bleeding rate of 15.7% in patients with HM3 [15,47]. Another nine-year retrospective study with three to six months follow-up on HM3 reported gastrointestinal bleeding in 10.4% of 48 implants [48].

A retrospective single-center study from the Mediterranean Institute for Transplants and Highly Specialized Therapies (ISMETT) reported that out of a total of 39 patients and over a mean period of 14 months, 7.7% of patients with HM3 presented gastrointestinal bleeding [37]. Data from the Momentum 3 study demonstrate that gastrointestinal bleeding occurs at a rate of 12-20% over one to two years after implantation, for 50 patients with BTT [38]. INTERMACS 2023 reported a rate of gastrointestinal bleeding of 8%, over a five-year follow-up with patients implanted with HM3 (10,920 patients) [18].

Multifactorial Causes

The presence of continuous flow can cause the onset of Von Willebrand disease, hypoperfusion of the gastrointestinal tissues with angiodysplasia; the same antiplatelet drug therapies can lead to a higher risk of hemorrhage. These events are more frequent in the female sex, a low level of hemoglobin in the blood before the implantation procedure, as well as low levels of International Normalized Ratio post-implantation, in individuals who smoke or are of advanced age [49]. There does not appear to be a direct relationship between the onset of gastrointestinal hemorrhages and the number of deaths [15]. The Heart Failure Association of the European Society of Cardiology reports that only 2% of deaths are linked to gastrointestinal bleeding [49]. Bleeding of the gastrointestinal system occurs more frequently in the upper tract (35-50%), and less frequently in the lower tract (15-22%) [50].

Another adverse event recorded with HM3 implantation is stroke, although in a lower percentage compared to previous generation implants [51]. A study prospectively evaluated the incidence of stroke in patients with LVAD, of which 72 with HM3 and 42 with HVAD; during the hospitalization period, 4% and 7% suffered a cerebral infarction, while after one to five years, the incidence of stroke was 10% for HM3 and 14% for HVAD [51]. INTERMACS recorded an incidence of stroke (hemorrhagic and embolic) of 16% out of 9,489 implants, with a risk of occurrence of 4% in the first 30 days post-implantation, 9% after six months, and 14% in the first 365 days post-implantation [52]. In MOMENTUM 3, over two years with HM3 implantation, the risk of stroke was 5%, while in the ISMETT study it was 7% [37]. Compared to patients with HM2, the occurrence of stroke in patients with HM3 at six months after implantation is 3.3 times lower, and a reduction in deaths from stroke, with 9.5% of deaths (13,738 patients with HM3) compared to previous generations (12.5%) [11,12].

Gender and Age

Risk factors are female gender (hemorrhagic stroke), ongoing infections and hypertension, previous cardiac surgery, anticoagulant therapy, and advanced age (ischemic stroke) [12,15,49,53]. The causes are always multifactorial. The formation of thrombi, rheological causes (Von Willebrand syndrome) [49]. In patients with HM3 compared to those with HM2, a reduction in endothelial nitric oxide synthesis is found, with a resulting increased arterial stiffness, which is a risk factor for stroke in this patient population. There is decreased cerebral vasodilatory capacity during exercise (with improvement following rehabilitation), likely reflecting dysautonomia and an imbalance of activity toward the sympathetic system [54].

Infections

Another event that is not prevented by the new generation of LVADs is the presence of infections. A retrospective study (2014-2020) of 75 LVAD implants evaluated infections affecting the driveline area; the observed data showed that infections occurred in 34.7% of patients (67 patients), with a 60% infection findings related to the presence of *Staphylococcus aureus* [55]. Generally, the percentage of patients with driveline-related complications in new-generation LVADs ranges from 18% to 70%, and this percentage increases with increasing device support time [8,18,37,50,56]. The risk of infection onset increases between three and six months after implantation [56]. Five years after implantation and in 13,738 patients, the percentage of infections was 62% [11]. Other risk factors include obesity, advanced age, diabetes, previous chronic and dermatological diseases, substance abuse and alcoholism, and inadequate hygiene habits [15,56]. The most frequent infections are associated with *Staphylococcus aureus* and *Staphylococcus epidermidis*, Enterococcus, *Escherichia coli*, and various fungal organisms, including *Pseudomonas aeruginosa *[56]. An attempt has been made to divide complicated and uncomplicated infections since 2024, or according to whether they are VAD-specific (driveline, pump, and other), VAD-related (infections linked to surgery), and non-VAD infections (other body areas affected by infections) [15,56]. These infections negatively impact rehospitalization and mortality, and in particular, the occurrence of hemorrhagic stroke [10,15,50]. Despite this problem, there are no optimal solutions.

Thrombi

The formation of thrombi in the cannulas and/or pump in patients with HM3 is rare, except in a few case reports [37,38,49,57]. Another event that has been studied is the finding of external outflow graft stenosis (eOGS) in patients with long-term HM3 treatment, caused by the accumulation over time of acellular biodegradable debris with a gelatinous substance between the external outflow graft and the artificial tissue (polytetrafluoroethylene, Gore-Tex) covering the outflow graft near the pump [58-60]. There is disagreement regarding the incidence of mortality and eOGS. Episodes of HM3 device structural malfunction are extremely rare [11]. Other events that could be alarming and interpreted as HM3 malfunction are the presence of suction (often caused by dehydration, right ventricular dysfunction), and blood accumulation (thrombus) in 0-1% of patients [11,12,18,38,48,50].

An adverse event to note is the LVAD retention time. Beyond two years after implantation, BTT patients demonstrate worse survival after HTx, and more post-surgical complications, compared to patients with an implant retention time of less than two years [61]. Despite the occurrence of major adverse events, once the patient is clinically stabilized, they can benefit from a non-pharmacological and non-invasive treatment that appears safe in this patient population, such as CR, which can make positive clinical adjustments (Table 1) [12].

**Table 1:** The table briefly summarizes the major adverse events that may be encountered in patients with LVAD. This table was created by the author (Bordoni Bruno) of this article. LVAD: left ventricular assist devices

Major adverse events
Right ventricular dysfunction (RHF) or "late right heart failure"
Heart failure
Gastrointestinal bleeding
Stroke (female gender, hemorrhagic stroke; advanced age, ischemic stroke)
Infections
Formation of thrombi in the cannulas and/or pump

Cardiac rehabilitation for patients with LVADs

ISHLT reports that all clinically stable patients with LVADs can access CR, on a type C level of evidence [12]. Similarly, guidelines from the Heart Failure Association (HFA) of the European Society of Cardiology (ESC) support the utility of performing CR for patients with LVADs, although these recommendations are underutilized [62].

When the inpatient can move safely, the initial phase is defined as “early mobilization” (EM) or the post-acute phase, during which active and passive movements are performed with the assistance of healthcare personnel (nurses, physiotherapists, and occupational therapists) [62]. EM-CR can be started when the patient is stable, regardless of the date of implantation, for example, the day after surgery; the decision will depend on the evaluation of the interprofessional team [12,62]. The goals of CR are to improve functional status, independence, and an acceptable quality of life [63]. Currently, there are no specific guidelines for a structured CR pathway for patients with LVADs.

A recent study evaluated 39 patients implanted between 2019 and 2021 (25 with HM3) and CR immediately after intensive therapy and entry into inpatient rehabilitation. CR consisted of a 1 h session across six weekly sessions, for a period of four to five weeks [64]. Training included endurance (interval) and resistance training using a cycle ergometer and small weights. Workload was subjectively assessed using cardiopulmonary exercise testing (CPET). Preliminary functional assessments were performed at the end of the CR cycle for comparison, including the 6-min walking test (6-MWT) and measures of peripheral muscle strength [64]. No adverse events were recorded. The parameters tested before and after CR improved, with a global response in exercise tolerance.

Another recent study evaluated patients implanted from 2009 to 2023, again after intensive therapy and within inpatient rehabilitation, with 274 patients with LVAD, of which 96 had HM3; only one patient died in the CR phase, and 2.9% presented bleeding and stroke [3]. Rehabilitation was structured with a double session for 6 days a week, with the use of an exercise bike, free body exercises, and an unspecified “diaphragmatic exercise”. The duration of CR was subjective, based on clinical stabilization, with a mean of 33±18 days [3]. The workload, both as an entity and the strategy used to organize and decide the training, was not illustrated. The 6MWT, the value of the Barthel Index, and the EuroQol Visual Analog Scale (EQ-VAS) improved at the time of discharge.

An exploratory review identified studies published within a 10-year period (2013-2023) on CR for LVAD patients, analyzing seven studies and 226 implants, highlighting that rehabilitation is safe in this patient population [65]. The study does not specify the type of HM. A review of the most recent publication dates for the same article indicates two studies from 2020 and two from 2021. In a 2020 study with 34 patients, the CR time frame ranged from five to 13 weeks, with three training sessions per week (15 sessions in total) over five weeks; the training included endurance (high-intensity interval training, HIIT). The amount of HIIT work varied between 50% and 90% of oxygen consumption (4 min at high intensity and 4 min at moderate intensity for four sets) and from 40% to 80% of maximum power output (30 s at high intensity and 4 min at low intensity) [65]. Parameters related to meters covered improved with the 6MWT. Data from 2021 studies, involving a total of 91 facilities, indicate an average cardiac rehabilitation (CR) duration of approximately one month. Training included interval or continuous low-load endurance with a stationary bike or treadmill, with an intensity of 60-70% of maximum oxygen consumption, and resistance/strength training for major muscle groups with variable sets and 12 repetitions per exercise [65]. Improvements were mainly related to the 6MWT and psychological parameters.

A 2019 Korean study evaluated 15 implants, of which only four were continuous flow. Patients underwent active mobilization three days after surgery, and unspecified breathing-related exercises [66]. Unspecified CR continued inward rehabilitation until discharge, with a 12-month follow-up. Improvements (assessed based on NYHA class, ultrasound parameters, and psychological parameters) were recorded in the first three months, with no variations at 12 months.

What emerges is the fact that after a few months of improvements, as per clinical stabilization after heart failure, the improvements cease, and a constant stability to physical effort remains; furthermore, the peak oxygen consumption under effort (VO_2 _peak) assessed with CPET does not increase with CR, remaining at 50% of the predicted [67]. A 2018 study subjected 68 implanted patients to CR, of which only four with HM3; the training was carried out in three to five weeks, with five to seven weekly sessions, following endurance training (exercise bike) with interval or continuous mode without specifying the intensity, and resistance training with three series and 20 repetitions each (without specifying the intensity) [68]. VO_2 _peak was only 37% predicted after CR, with performance gains recorded in meters with 6MWT. Cardiopulmonary function remained stably reduced, and this finding was observed in both HM2 and HM3 patients [69]. The lack of increase in this finding was recorded after three months post-implantation and did not change even after 12 months post-implantation, regardless of the CR modality (high-intensity interval training {HITT}, continuous/interval endurance, resistance training), or the parameters used (sets, repetitions, time frame, number of sessions, resistances) [7,70,71]. When VO_2 _peak appears to undergo a clear improvement, and the characteristics of the patients are observed, it emerges that the initial condition before the implant was clinically severely depressed, or the data were not compared with control groups [72,73]. We recall that VO_2 _peak incorporates information such as peripheral oxygen utilization (using Fick's law), heart rate, and stroke volume, and that it reflects the patient's survival capacity with an LVAD [21,74].

IMT and Training Recommendations

**Figure 6:**
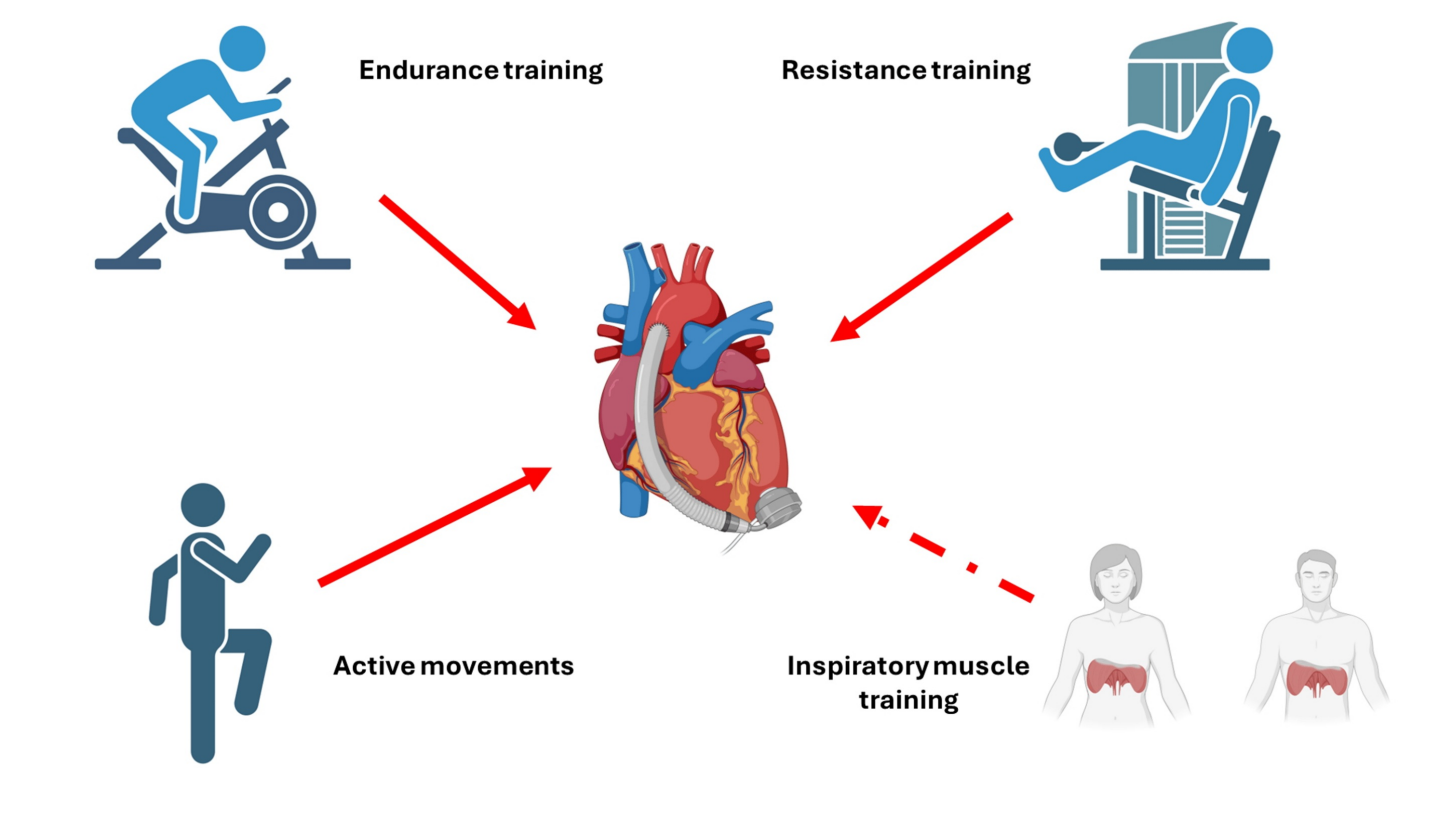
The figure schematically illustrates physical activity in the rehabilitation process, such as endurance training, resistance training, simple active mobilization, highlighting the fact that respiratory muscle training is not routinely added to cardiac rehabilitation in patients with LVAD implantation (HM3). This image was created by the author (Bordoni Bruno) of this article using BioRender.com. HeartMate 3 (HM3; Chicago, IL: Abbott) LVAD: left ventricular assist device

Another fact that emerges is an almost total absence of IMT within CR, and a complete lack of precise indications regarding the organization of IMT (sets, repetitions, and resistance) (Figure 6) [7,70-74]. HFA and ESC suggest managing the load to be used for the patient (endurance and/or resistance training), by previously performing CPET or 6MWT [62]. If the values ​​derived from these two tests indicate VO_2 _peak >14 mL/kg/min for CPET and/or meters covered >300 for the 6MWT, the clinician may implement the patient's workload [62]. For every 10 meters below the threshold of 300 meters, the mortality rate increases by 21% [69].

However, there is no gold standard for endurance and resistance training in patients with LVADs, nor for IMT parameters in this category of patients. Endurance and resistance training administered to patients typically yields better results with submaximal, low-impact workloads, such as 65% of VO_2 _max [11,69]. The patient's daily activity focuses on submaximal performance; furthermore, the pump speed does not satisfy the peripheral demands of maximal muscular activity, generating hypoperfusion during any training [70]. The intensity of the training must always be subjectivized to the patient's work capacity, so as to always make CR safe [75]. Some authors recommend using the rating of perceived exertion (RPE) during CR, with an activity that includes an RPE value between 11 (light) and 13 (somewhat hard); they also recommend using the Talk Test to avoid situations of apnea [73]. Other authors recommend using RPE between 12 and 14 (quite heavy) [12].

What happens to patients with LVADs and CR?

With training, LVAD patients improve their functional status and exercise tolerance compared with their pre-implant HF condition; their quality of life also improves [11,70]. Cardiac output, regulated by pump speed, intrinsic left ventricular contractility, and reduced peripheral resistance, increases the amount of oxygen delivered to the periphery and its extraction [70].

LVADs unload the workload of the left ventricle and its oxygen consumption; increased cardiac and peripheral oxygen extraction decreases the amount of circulating N-terminal pro-B-type natriuretic peptide (NT-pro BNP), which is inversely correlated with meters walked during the 6MWT [69]. This effect is most evident during the first six months, after which NT-pro BNP values ​​and meters walked remain stable.

CR improves endothelial function, decreases the progression of coronary atherosclerosis, and improves emotional status (anxiety and depression) [72]. Endurance and resistance training improve cerebral blood perfusion in patients with HM3 and, likely, brain function as well [76]. Right ventricular contractility appears to improve with submaximal efforts but does not appear to increase with greater efforts [21]. Lean mass and strength increase, especially in the first six months of CR, demonstrating an inverse relationship with NT-pro BNP values, the cytokine growth differentiation factor 15 (GDF-15), and high-sensitivity C-reactive protein (hsCRP) [77]. If the myocardium has recovered sufficiently, it can be removed from the LVAD, albeit with a low percentage (0.7% at two years) for BTT patients, while for BTR patients, the percentage is approximately 5% [78,79].

Diaphragm and HF

There are several reasons why greater attention should be paid to the diaphragm muscle in patients undergoing a CR procedure, foremost among them, diaphragmatic weakness. If the patient's path with implant derives from HF, we know that the skeletal muscles have undergone non-physiological morphological, structural, and functional adaptations. For example, the limb muscles are hypotrophic with a phenotypic change from aerobic fibers (red or type I) to anaerobic fibers (white or type II), there is a decreased capillary organization, with increased apoptosis, there is an increase in fat and connective tissue within the muscle fibers, a reduced sarcoplasmic reticulum, and a disorganization of the sarcomeric structure [19,80,81].

These myopathic adaptations, not always fully understood, may result from an imbalance of the immunological and neurological systems, with chronic systemic inflammation and constant activation of the sympathetic nervous system [82]. During physical activity, this condition generates abnormal afferent signaling via type III and IV muscle afferents, leading to increased sympathetic nervous system activation. Through multiple mechanisms, this response results in fatigue and exercise intolerance, with a consequent reduction in VO_2 _peak (exaggerated mechano-metaboreflex). This vicious cycle underlies the “muscle hypothesis” [19].

Similarly, the diaphragm changes its performance capacity, and at a faster rate than the periphery [19]. The neuromuscular endplate undergoes structural alterations, including partial denervation, resulting in a reduction in contractile capacity of approximately 15-30% compared with healthy individuals, along with a 20-30% decrease in contraction speed. These adaptations reflect a reduction in certain sarcomeric proteins, such as myosin heavy chain (MHC) and titin, and are associated with thinning of the diaphragm [19]. The diaphragmatic muscle fiber is weak and performs poorly. Contrary to the myopathic adaptation that occurs in the muscle fibers of the limbs, the diaphragm has a phenotypic change towards an increase in red fibers (always inefficient), and a decline in white fibers [83].

The diaphragm is less strong, with a 30% decrease in maximal inspiratory pressure (MIP), reaching the fatigue threshold more quickly compared to healthy subjects [83]. Furthermore, the movement expressed by the diaphragm is reduced (less excursion), and this event correlates directly with the NYHA class and with an inverse relationship with the pulmonary artery systolic pressure [19,84]. Diaphragmatic fatigue reflects the decrease in peak VO_2_ and the finding of dyspnea [19]. In patients with HF, the thickness of the diaphragm (decreased thickness) reflects the decreased strength of the limbs, a decreased value of the forced expiratory volume in 1 s (FEV1); diaphragmatic hypotrophy is directly linked to an increase in mortality in patients with HF and to the increase in blood BNP values ​​[85]. A dysfunctional diaphragm may be the cause of a restrictive pattern on spirometry, but it does not truly reflect the health status of the lungs [86]. Symptoms related to intolerance to physical activity are not necessarily associated with instrumental measures of cardiac (e.g., ejection rate) or pulmonary function but may be associated with diaphragmatic dysfunction [87-89].

**Figure 7:**
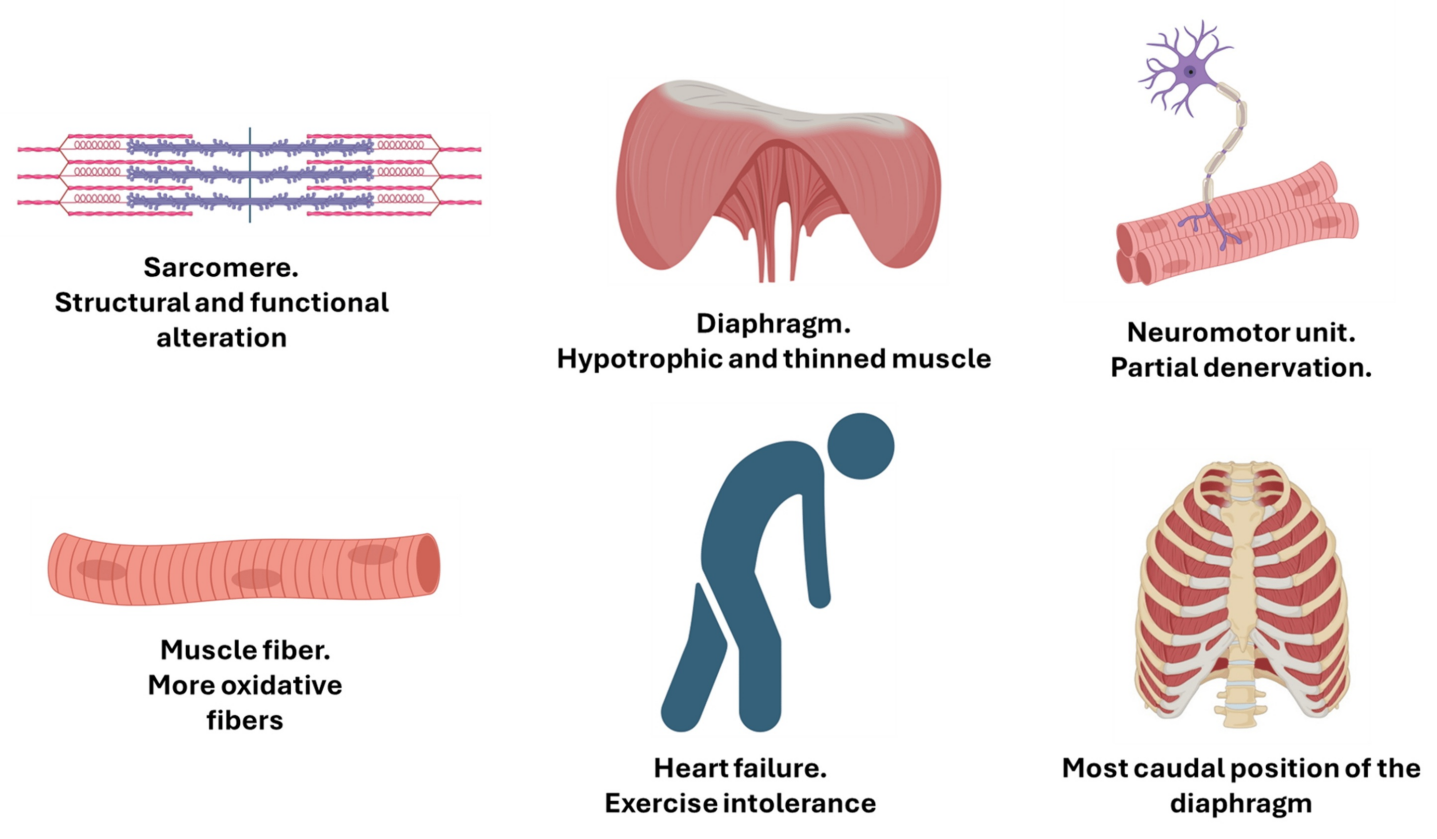
The image illustrates the myopathic adaptations observed in patients with heart failure, highlighting structural and functional alterations of the diaphragm muscle. Diaphragmatic dysfunction can induce exercise intolerance. This image was created by the author (Bordoni Bruno) of this article using BioRender.com.

The diaphragm in HF patients has an inspiratory resting attitude (displaced towards caudality) with shortening fibers; this adaptation further causes a decrease in expressed force and a reduced intrapleural pressure [88]. This preferential position towards caudality could be the result of a larger ventricle; there is a close relationship between the size of the left ventricle and the position (and therefore the diaphragmatic force) of the diaphragm [88]. During inspiratory acts against resistance (as with IMT), the excursion of the diaphragm increases and becomes comparable with healthy subjects [88]. Finally, a faster but more superficial contraction velocity is observed in patients with HF compared with healthy subjects [88]. Decreased diaphragmatic thickness correlates with lung function in patients with HF; reduced strength is associated with smaller lung volume [90]. Non-physiological changes in lung volumes will adversely affect myocardial flow and pressure (Figure 7) [91].

Diaphragm and LVAD

The cumbersome presence of the pump above the diaphragm influences the position of the muscle, which will be more predisposed to assume a caudal position [92]. The more caudal the diaphragm, the greater the likelihood of pump dysfunction, which is also attributable to suboptimal surgical implantation techniques [92,93]. It is plausible that these positional adaptations could be one of the causes leading to further cardiovascular failure in patients with long-standing HM3. Specifically, not only does the left ventricle lose its intrinsic contraction capacity over time, but the caudal position of the diaphragm could further stretch the ventricular fibers, causing them to lose strength. We still have no precise answers regarding the onset of HF in patients with long-standing HM3, particularly if the flow velocity is increased over time by the clinician out of necessity (ventricular atrophy?); MOMENTUM 3 reveals that 20% and 30% of patients experience unexpected ventricular deterioration after two and five years, respectively [94].

Cardiac surgery itself (and possible subsequent assisted ventilation) weakens the diaphragm, likely due to phrenic nerve involvement, regardless of LVAD placement. Patients undergoing cardiac surgery suffer from diaphragmatic dysfunction in 60-75% of cases, although the causes are not always identified [95,96]. Generally, patients with post-surgical diaphragmatic dysfunction develop damage on one side of the heart (right or left have similarly the same percentage), and only 7.6% are symptomatic [95]. This means that most patients who do not undergo instrumental tests to verify the position/functioning of the diaphragm 96 h after surgery (the maximum time for spontaneous recovery of the diaphragm) may have dysfunctions that are misdiagnosed as other clinical signs (dyspnea, fatigue, exercise intolerance) [95,97]. Diaphragmatic dysfunction may persist for a long time after thoracic surgery (up to one year), and not all patients recover [98]. Post-surgical diaphragmatic dysfunction may involve LVAD patients in approximately 38% of cases, but this percentage is underestimated [99].

After LVAD implantation, although some cardiovascular parameters improve, lung function/efficiency parameters (spirometric data) do not [100]. A decline in lung diffusion (alveolar-capillary diffusion) has been reported, and, as previously noted in the article, VO_2 _peak does not increase by a substantial percentage and is independent of gender [30,101-103]. VO_2 _peak derived from CPET is related to the difference between the diaphragm excursion during inspiration and expiration [104]. This would reflect the weakness of the diaphragm in LVAD patients, but we have no data to support this hypothesis.

As mentioned, spirometric data from implanted patients are limited. A 2020 meta-analysis reported that, across 219 implants (26% with HM3), forced expiratory volume in 1 s (FEV1) and forced vital capacity (FVC) were reduced, as was global lung diffusion capacity [105]. These data provided insight into a restrictive lung pattern, but the negative results obtained with spirometry may derive from an alteration of the diaphragm in this patient population [105]. Therefore, we can infer that a small percentage of LVAD patients are diagnosed with a certain diaphragmatic dysfunction, while the majority were not directly diagnosed with specific instrumental tests (echocardiography, Doppler, X-rays, fluoroscopy), despite the decrease in respiratory function. Further data are needed, especially for long-term adaptation [105]. It should be remembered, however, that unlike cardiothoracic surgery, the positioning of the LVAD requires an incision of the diaphragm from its subdiaphragmatic portion, and this lesion remains for life [106].

Another consideration is VO_2 _max and patients with LVAD. VO_2 _max after 12 months remains below the predicted percentage values ​​for sex and age, and this value of oxygen consumption depends on cardiac output and oxygen extracted from skeletal muscles [69]. It should be remembered that the diaphragm is richly supplied with blood, thanks to multiple vascular pathways, such as the phrenic arteries, internal thoracic arteries, and intercostal arteries. These blood pathways form structures that function independently of one another; this helps ensure that an adequate amount of blood reaches the diaphragm [107].

Blood flow correlates with the contractile capacity of the diaphragm; an increase in blood flow to the inspiratory muscle will improve its excursion (in patients with CHF) [90]. We could hypothesize that during an effort, the blood flow to the diaphragm increases, while the amount of blood reaching the limb muscles is reduced (as in an animal model); this would reflect the preferential phenotype with HF (larger red fibers for the diaphragm and larger white fibers for the limbs) [108]. Furthermore, the VO_2 _max value may better represent diaphragmatic work than peripheral muscle work in patients with implants, owing to a greater blood supply. These hypotheses still need to be verified.

We know that the diaphragm preferentially stimulates the intervention of the parasympathetic system, through the activation of various body receptors (somatic and visceral) during inspiration, through spino-solitary and spino-trigeminal pathways; the responses that follow inspiration are an increase in the vagus nerve and a decrease in the sympathetic system [109-112]. We know that patients with LVAD tend to have a more active sympathetic nervous system, despite hemodynamic improvements, particularly if there is a previous history of HF, as happens for HF patients without LVAD implant [113-115]. We could hypothesize that a neurohormonal dysregulation could derive from a diaphragmatic dysfunction, which, in the presence of LVAD, is not able to effectively stimulate the response of the parasympathetic system, as it lacks sufficient contractile force. Another hypothesis that requires further investigation.

IMT and LVAD

The first article evaluating patient adaptation with LVAD and IMT dates to 2006, a case report of BTT. IMT was started approximately 20 weeks after implantation, with a device that allowed the use of an inspiratory resistance to 60% of maximal inspiratory pressure (PImax) across three weekly sessions and 10 weeks of training [116]. Several improvements were observed, including VO_2 _peak and lung volumes. It is not clear, however, whether IMT alone, or in combination with other training modalities integrated into a CR program, should be credited for such positive clinical adaptations. Another 2011 study followed 15 patients with BTT implants who underwent CR (three to five sessions per week for 10 weeks) with IMT; the latter was organized as training until exhaustion, with three sessions for 10 weeks, with PImax of 60% [117]. Some functional parameters, including 6MWT and VO_2 _peak, improved but did not differ significantly from the control group, whereas inspiratory force (PImax) improved, with benefits for the sensation of dyspnea. The study did not specify why that specific resistance was chosen for IMT in patients with LVAD. Including IMT in a CR program for LVAD patients appears to be free of complications [30,118]. We do not know how the diaphragm adapts to the presence of an LVAD, and what the real consequences of a non-physiological adaptation are. Currently, there are no guidelines for IMT in this patient population.

We can hypothesize that training the diaphragm and patients with an LVAD is based on a more rational and targeted approach. In healthy subjects, the IMT session should not exceed 30 min of work (always with a pause between one inhalation and the next), as beyond this time, muscular fatigue inevitably sets in [119]. The workload should not be intense (over 85% of VO_2 _max, and over 60% of PImax), as fatigue sets in after a few minutes (2-3 min) of resisted inhalations, with activation of the sympathetic nervous system and peripheral vasoconstriction (in patients without LVAD) [119,120]. Furthermore, if diaphragmatic fatigue sets in, 5-30 minutes of rest may be necessary for full recovery; when the diaphragm becomes fatigued, the expiratory muscles also experience a decline in performance [119].

Regarding repetitions and sets for healthy subjects, the American College of Sports Medicine (ACSM) provides recommendations based on training objectives, taking into account fiber type recruitment (white {type II} or red {type I} fibers). If it is necessary to recover the functionality and number of white fibers, it is recommended to perform resistance training that includes loads to be overcome/lifted corresponding to 45-50% of the subjective capacity to lift a load only once (one repetition maximum {1RM}), while for people who follow a routine training, the load moves to 60-70% of the 1RM; a number of repetitions of approximately 8-12, a number of sets per muscle group that is approximately one to three (two to four sets for more trained people), respecting a rest between one set and the other of 2-3 min before resuming the next set, with a weekly organization of 2-3 sessions [121]. It is possible to increase the resistance to be overcome by 2-10% if the training load starts to become easier to follow.

For training that specifically targets red (type I) fibers, or endurance training, the ACSM recommends a workload of 40-60% of one-repetition maximum (1RM), with 10-15 repetitions per set (15-25 repetitions for trained individuals) and multiple sets (number not clearly specified). Rest periods between sets should be approximately one minute (or 90 seconds for trained individuals), and sessions should be performed two to three times per week (two to four times for trained individuals) [121]. If the load to be overcome becomes too light, it can be increased subjectively.

As already noted in another article on HF, PImax (a value derived from a pressure gauge) could represent 1RM [19]. We could hypothesize that, to stimulate diaphragmatic white fibers, it is possible to use a PImax of 50-60%, performing one to three sets of eight to 12 inspirations (with a pause between each breath), with a rest interval of approximately 2-3 min between sets, and a total of no more than three sessions per week [19]. For diaphragmatic red fiber stimulation, a subjective PImax of 40-50% could be used, with 10-15 inspirations per set, performed over one to three sets, a rest interval of approximately 1 min between sets, and a maximum of two to three sessions per week [19].

We know that submaximal physical activity (resistance and endurance training) achieves greater results in patients with LVADs. IMT work should probably be submaximal. It should be noted that diaphragmatic fatigue reduces limb strength in healthy subjects and that diaphragmatic fatigue in patients with LVADs could probably negatively impact the skeletal muscles' strength [122].

The management of IMT sessions should be integrated into the usual CR program, which includes active movements, cycling/treadmill, and strength training, without exceeding the intensity of the work. For weaker patients or those who are not yet able to move independently, using an initial CR approach with IMT could be a useful strategic choice (as in our clinical practice). Considering the previous diagnosis of HF in many patients and the diaphragmatic weakness that reflects pulmonary function, some authors recommend starting IMT as soon as possible [123]. Following IMT improves the NYHA class in patients with HF [124].

**Figure 8:**
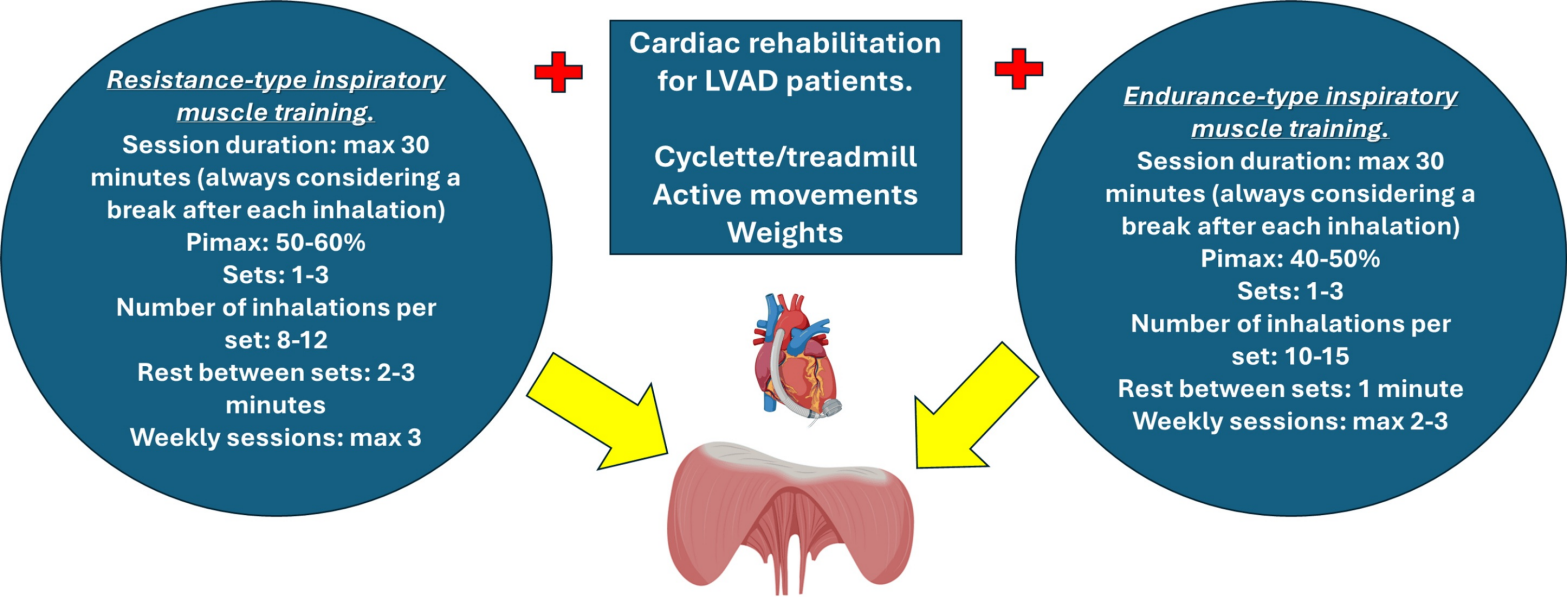
The image illustrates parameters that could be used for inspiratory muscle training (IMT). This image was created by the author (Bordoni Bruno) of this article using BioRender.com.

There appears to be a gender-based difference in fatigue under normoxia. Women reach fatigue more slowly than men, with a greater endurance capacity [125]. In the presence of acute hypoxia, on the contrary, women experience fatigue earlier than men [126]. We do not know whether this difference is found in patients with LVAD (Figure 8).

Future challenges

In clinical practice (in non-implanted patients), diaphragmatic dysfunction is often underestimated, and instrumental tests are not always performed to assess its function, despite the countless causes that alter its structure [127]. Patients with a single-sided lesion are more difficult to detect, as they are asymptomatic at rest [127]. There are multiple concomitant causes that lead to diaphragmatic dysfunction (in non-implanted patients), including sepsis, systemic inflammation, acidosis, steroid therapy, malnutrition, ionic metabolic alterations, obesity, statins (statin-associated muscle symptoms), previous cardiac surgery, and myocardial infarction [127-129].

Research should focus more on diaphragm rehabilitation in this patient population and on determining the potential non-physiological adaptations that occur in this muscle with LVADs. Another key point is diaphragm assessment, which should become routine, not only to identify potential dysfunctions and obtain additional information to adjust and personalize IMT, but also to identify possible causes of symptoms (dyspnea, fatigue, exercise intolerance), thereby improving the assessment of the patient. There is a lack of guidelines for IMT, not only for the systematic implementation of such training, but also how to insert it into other physical activities already present in the CR; furthermore, we do not know what is the correct rest that the patient should follow between one session and another, to avoid the so-called overtraining syndrome (OTS), with a reduction in the expected clinical improvements. OTS is the failure to improve performance, with a decline in the same, as the person has not been able to adequately recover from the previous training session, resulting in the accumulation of fatigue [130]. ​​It follows that the intensity of exercises in the CR, including IMT, should not always have the aim of using incremental loads at each session, but it should include sessions in which the load is lower than the previous training.

## Conclusions

The growing aging population, improved care, and the inevitable increase in patients with end-stage chronic heart failure (HF) have created a disparity between the availability of heart transplantation (HTx) and the number of patients requiring a new heart. To address the discrepancy between the need for and shortage of HTx and to prolong patient survival, durable mechanical circulatory support (DMCS) device technology has advanced significantly, providing clinicians with greater therapeutic options. The most implanted LVADs are durable left ventricular assist devices (LVADs), with the new-generation Heart Mate 3 (HM3, Chicago, IL: Abbott) demonstrating a reduced mortality rate five years after implantation.

Patients with LVADs can safely participate in cardiac rehabilitation (CR) and achieve clinical improvements through aerobic and anaerobic exercise. The literature is sparse on studies integrating diaphragm training, such as resistance exercises during inspiration or IMT, into CR programs. Some data suggest paying greater attention to diaphragm function in patients with implants and routinely incorporating IMT into the CR program. This article has raised new considerations regarding the parameters to be followed during IMT, highlighting the importance of achieving a better-performing diaphragm in patients with LVADs.
